# Risk factors for VIA positivity and determinants of screening attendances in Dar es Salaam, Tanzania

**DOI:** 10.1186/1471-2458-12-1055

**Published:** 2012-12-07

**Authors:** Crispin Kahesa, Susanne Kruger Kjaer, Twalib Ngoma, Julius Mwaiselage, Myassa Dartell, Thomas Iftner, Vibeke Rasch

**Affiliations:** 1 Department of International Health, Immunology and Microbiology, University of Copenhagen, Copenhagen, Denmark; 2 Ocean Road Cancer Institute, Dar es Salaam, Tanzania; 3 Department of Virus, Lifestyle and Genes, Institute of Cancer Epidemiology, Danish Cancer Society, Copenhagen, Denmark; 4 Gynecologic Clinic, Rigshospitalet, University of Copenhagen, Copenhagen, Denmark; 5 Department of Experimental Virology, Universitaetsklinikum, Tuebingen, Germany; 6 Department of Gynecology and Obstetrics, Odense University Hospital, Odense, Denmark

**Keywords:** Cervical cancer, Screening, VIA, HPV, HIV, Tanzania

## Abstract

**Background:**

Tanzania is among the countries in the world where the cervical cancer incidence is estimated to be highest. Acknowledging an increase in the burden of cervical cancer, VIA was implemented as a regional cervical cancer screening strategy in Tanzania in 2002. With the aim of describing risk factors for VIA positivity and determinants of screening attendances in Tanzania, this paper present the results from a comparative analysis performed among women who are reached and not reached by the screening program”.

**Methods:**

14 107 women aged 25–59 enrolled in a cervical cancer screening program in Dar es Salaam in the period 2002 – 2008. The women underwent VIA examination and took part in a structured questionnaire interview. Socioeconomic characteristics, sexual behavior, HIV status and high-risk (HR) HPV infection were determined in a subpopulation of 890 who participated and 845 who did not participate in the screening.

**Results:**

Being widowed/separated OR=1.41 (95% CI: 1.17-1.66), of high parity OR=3.19 (95% CI: 1.84-5.48) of low education OR= 4.30 (95% CI: 3.50-5.31) and married at a young age OR=2.17 (95% CI: 1.37-3.07) were associated with being VIA positive. Women who participated in the screening were more likely to be HIV positive OR= 1.59 (95% CI. 1.14-2.25) in comparison with women who had never attended screening, while no difference was found in the prevalence of HR-HPV infection among women who had attended screening and women who had not attended screening.

**Conclusion:**

Women who are widowed/separated, of high parity, of low education and married at a young age are more likely to be VIA positive and thus at risk of developing cervical cancer. The study further documents that a referral linkage between the HIV care and treatment program and the cervical cancer screening program is in place in the setting studied, where HIV positive were more likely to participate in the cervical cancer screening program than HIV negative women.

## Background

With an estimated 530,000 new cases annually, cervical cancer is the third most common cancer in women and has to be considered a major health concern worldwide [1]. More than 85% of the global burden of cervical cancer occurs in developing countries. The high-risk regions are Eastern and Western Africa. In these regions the age standardized incidence rate is greater than 30 per 100,000. In comparison, the age standardized incidence rate in North America and Australia/New Zealand is less than 8 per 100,000. Cervical cancer is responsible for 275,000 deaths annually, about 88% of which occurs in developing countries [1]. One of the explanations behind the high mortality in developing countries is the lack of effective screening programs for cervical cancer. As a consequence, no clinically significant reduction in the incidences of cervical cancer has occurred [2,3]. In contrast, there has been a major decline in cervical cancer mortality in developed countries after the introduction of population-based screening programs based on Papanicolaou smears (Pap smears) to detect cervical abnormalities [4].

A screening program based on Pap smears requires several steps, including health care providers who are able to collect the cells from the cervix and prepare slides, cytopathologists who can stain and read the slides and a communication network that can convey the results to the women and arrange for a second visit if the smear is abnormal. The infrastructure required for these steps is unavailable in most developing countries. Therefore there is a need for more simple screening methods which can be interpreted immediately and combined with treatment. Visual inspection of the cervix after acetic acid application (VIA) is believed to be an effective method for screening in resource-limited settings [5]. VIA is performed by a trained health care provider who applies acetic acid solution to the cervix and then observes the transformation zone of the cervix for 1 to 2 minutes for acetowhite epithelium, which is thought to be indicative of abnormal cellular changes [6]. The implementation of VIA as a screening strategy has shown promising results and screening programs based on this methodology are being implemented increasingly in low income countries [7,8].

Tanzania is, with an age-standardized incidence rate of 50.9 cases per 100,000 women, and an age-standardized mortality rate of 37.5 per 100,000 women due to cervical cancer, one of the countries in the world hardest hit by the disease. The high burden of disease is plausible due to high prevalence of HPV and HIV. Women who are HIV-positive are more likely to develop cervical cancer than HIV-negative women [9]. With an HIV prevalence rate of 9% among women aged 15–49, this has significant implications for the cervical cancer screening program [10].

Acknowledging an increase in the burden of cervical cancer, VIA was implemented as a regional cervical cancer screening strategy in 2002. The initiative was supported by the World Health Organization (WHO) and the International Agency for Research on Cancer (IARC). These organizations assisted Ocean Road Cancer Institute (ORCI), the national cancer hospital in Tanzania, in implementing a national cervical cancer screening program, based on VIA, for the detection of precancerous lesions. The program targeted women aged 25–59 years and initially used acetic acid and Lugol’s iodine to evaluate the comparative accuracy of the tests [7]. The initial project ended in 2005 and cervical cancer screening services have since then been routinely available in a few satellite sites, including Dar es Salaam, Kigoma, Kilimanjaro and Morogoro. Since 2005, efforts have been made to scale up the service to regional and district hospitals through training of health providers and by integrating the service in the reproductive and child health clinics and/or gynecologic clinics. The service is offered free of charge and is done by trained doctors and nurses. With the aim of describing risk factors for VIA positivity and determinants of screening attendances in Dar es Salaam, Tanzania, this paper present the results from a comparative analysis performed among women who are reached and not reached by the screening program.

## Methods

### Study population and enrollment

The present study enrolled a subgroup of women aged 25–59 years, who had participated in the National Cervical Cancer Screening program at ORCI between November 2002 and December 2008. In this period, 15,439 women were screened for cervical cancer. In all, 1246 women aged either below 25 or above 59 years and 86 women with incomplete forms were excluded from the study, leaving 14,107 women. Of these, 932 women were diagnosed as having a positive VIA test and the remaining 13,175 as having a negative VIA test.

To evaluate whether women reached by the screening program differed from those not screened, all women who participated in the screening program from February to June 2008 were compared with a reference group of women who had never participated in the screening program. The reference group was recruited from three municipalities of Dar es Salaam. To identify a representative group of women who had never attended a screening, a random sample of 1500 houses was selected from the national house hold survey [11] using multistage cluster sampling where three wards from each of the three municipalities in Dar es Salaam were randomly selected and from each ward five streets were randomly selected to ensure equal representation from the three districts. The recruitment was done between February and June 2008 by a research assistant who worked as a social worker. The community leaders, who were responsible for the sampled streets, assisted in the recruitment of the women who were informed about the project via home visits and through megaphone campaigns. Health information was provided through the campaigns and the women were invited to attend outreach screening for cervical cancer at the nearest municipal hospital. In all, 1599 women were found to be eligible for the study and a total of 845 women aged 25–59 were enrolled. All women who were enrolled in the study were interviewed by the principal investigator and a trained research assistant.

### Data collection

#### VIA status and socioeconomic and reproductive characteristics

All women participating in the screening program were screened by means of VIA, which was performed by a nurse provider. VIA test was used instead of VILI due to 1) Easier preparation of acetic acid versus Lugol’s iodine and; 2) Higher specificity of VIA compared to VILI which reduces the risk of false positive results.

In addition, information on socioeconomic and reproductive characteristics was collected using a structured questionnaire. Since the screening staff at ORCI had been trained in VIA and were experienced in using VIA to detect precancerous lesions, this test was used rather than the colposcopic examination.

#### Cervical cancer risk factors and screening attendance

The 890 women from the sub population of women participating in the screening program and the reference group of 845 non-screened women were interviewed about their sexual behavior, general health condition, STIs, and circumcision of male partners.

Cervical samples were collected and placed in a transport medium and kept frozen at −18°C. The samples were kept at ORCI for a maximum of two months and then stored on dry ice and shipped to Germany (Tuebingen University) and tested for HR-HPV by means of the Hybrid Capture 2 (HC2 Qiagen, Hilden, Germany) high-risk (HR) probe.

VIA was performed according to WHO/IARC protocol. Acetic acid was applied to the cervix using a cotton swab and the cervix was visualized after a minute with the aid of a halogen light source. The test was classified positive if acetowhite epithelium appeared near the Squamous Columnar Junction. The test was classified suspicious for cancer if a cauliflower-like fungating mass or ulcer was noted on the cervix. If the cervical lining was smooth, uniform, with no acetowhite lesion the test was classified as negative [12].

After the gynaecological examination, the women were offered voluntary counseling and testing for HIV. HIV status was determined using the National algorithm for HIV testing [13]. The serological test was based on the Trinity Biotech’s Uni-Gold™ Recombigen® HIV test. If the test result was positive a repeat serological test was performed utilizing Abbott Determine HIV-1/2. This was done to avoid false positive results.

#### Ethics

Permission to carry out the study was obtained from the National Institute of Medical Research (NIMR) in Tanzania and the Danish National Committee on Biomedical Research Ethics. Informed consent to participate in the study was obtained from each participant. Women who were found to have pre-cancerous lesion were treated by cryotherapy or LEEP based on disease grade. Similarly those who were found to have invasive cervical cancer were treated with radiotherapy as per ORCI protocol. Women who tested HIV positive were assisted to attend the HIV care and treatment clinic. All services were provided free of charge.

#### Statistical analysis

Data were entered in EPIINFO version 6.04 and then exported to SPSS version 13 for analysis. Odds ratios (ORs) and 95% confidence intervals (CI) were computed as a measure of association between socioeconomic and reproductive characteristics and VIA status. Similarly the association between previous screening attendance and socioeconomic characteristics, sexual behavior, HIV status and HR-HPV infection were assessed by ORs. Multiple logistic regression analysis was used to adjust the risk estimates. All variables (age, education, parity, HIV status, HR-HPV, age of sexual intercourse and number of sexual partners) that were statistically significant at a p value of 0.2 or less in the bivariate analysis were considered for multivariate analysis. Final variable selection was done using a backward stepwise elimination process. A P value of ≤ 0.05 was considered significant. Multivariate logistic regression was also used to assess for interaction and confounding. We used the chunk test to see if there was interaction by comparing the -2LL (negative two log likelihood) of the reduced model (no interaction terms) and the full model (with interaction terms). We found no significant difference between these two models and concluded that there was no interaction. All variables were tested for confounding since they were not effect modifiers. We noted the ORs for HIV and HR-HPV when each of the variables was left out of the model and also when it was put back in the model. The variables that changed the OR of HIV-1 and HR-HPV by more than 10% were considered confounders. The variables were tested singly and also as groups of confounders. All statistical tests were two-sided. We also performed stratified analysis of HR-HPV and HIV infection among screened and unscreened women.

## Results

Table 1 shows the socioeconomic characteristics of 13,217 women attending cervical cancer screening in 2002–2008 and a subpopulation of 890 women who participated in the screening program between February and June 2008. The subpopulation seemed to be older and of lower parity.

**Table 1:** Comparison of socioeconomic characteristics of women attending cervical cancer screening (2002-Jan 2008) and a subpopulation of women who participated in screening between February and June 2008

**Variable**	**Participated in the screening program N=13,217**	**Subpopulation of women who had participated in the screening program N=890**	**p-value**
**Age**			
25-34	4552(34.4)	259(29.1)	
35-44	5028(38.0)	340(38.2)	0.01
45-59	3637(27.5)	291(32.7)	
**Parity**			
0	536(4.0)	79(8.9)	
1-2	3671(27.8)	307(34.5)	
3-4	4041(30.6)	293(33.0)	< 0.001
≥ 5	4457(33.7)	210(23.6)	
Missing	512(3.9)		
**Educational Level**			
No formal education	1172( 8.9)	74(8.3)	
Primary school	7118( 53.9 )	529(59.4)	0.05
Sec school and above	4915( 37.2 )	287(32.2)	
Missing	12(0.1)		
**Marital Status**			
Married	10801(81.7)	732(82.2)	0.16
Single	542(4.1)	37(4.2)	
Widowed/Separated	1849(14.0)	115(12.9)	
Missing	25(0.2)	6(0.7)	

Table 2 shows the determinants of VIA status among women in Dar es Salaam. Seven pct. (932/14,107) of the women screened were found to be VIA positive. In the bivariate analysis, women aged 45–59 had an increased crude OR of 1.78 (95% CI: 1.51-2.41) for being VIA positive in comparison with women in the younger age group (25–34 years). In the age adjusted analysis, women who had never attended school or only attended primary school had statistical significantly increased ORs of 4.30 and 1.58, respectively, for being VIA positive compared with women who had attended higher level education. The women’s marital situation was also associated with being VIA positive, more specifically widowed/separated women had an increased OR of 1.41 (95% CI: 1.17-1.66) for being VIA positive in comparison with married women. Moreover, women who were married at a young age (14–18 years) had an increased OR of 2.17 (95% CI: 1.37-3.07) for being VIA positive in comparison with women who were married at age 29 or above. Finally, women who had 1–2 children, 3–4 children and 5 or more children had increased ORs of 2.38, 2.43 and 3.19, respectively, for being VIA positive in comparison with women who had no children.

**Table 2:** Determinants of VIA positivity among women in Dar es Salaam, Tanzania

**Variable**	**VIA positive N=932**	**VIA negative N=13175**	**OR (95% CI)**	**Age-adjusted OR (95% CI)**
**Age**				
25-34	256(27.5)	4555(34.6)	1	
35-44	319(34.2)	5049(38.3)	1.11(0.94-1.33)	
45-59	357(38.3)	3570(27.1)	**1.78(1.51-2.41)**	
**Educational Level**				
No formal education	217(23.3)	1029(7.8)	**4.87(4.04-6.03)**	**4.30(3.50-5.31)**
Primary school	499(53.5)	7148(54.2)	**1.64(1.45-1.91)**	**1.58(1.33-1.87)**
Sec. school or above	213(22.8)	4989(37.9)	1	1
Missing	3(0.3)	9(0.1)		
**Marital Status**				
Married	710(76.2)	10823(82.1)	1	1
Single	22(2.4)	557(4.2)	**0.64(0.42-0.93)**	1.05(0.66-1.62)
Widowed/Separated	198(21.2)	1766(13.4)	**1.71(1.44-2.02)**	**1.41(1.17-1.66)**
Missing	2(0.2)	29 (0.2)		
**Age at Marriage**				
14-18	362(38.8)	3228(24.5)	**2.32(1.62-3.34)**	**2.17(1.37-3.07)**
19-23	351(37.7)	5271(40.0)	1.38(0.96-1.98)	1.31(0.83-1.92)
24-28	139(14.9)	3075(23.3)	0.94(0.64-1.38)	0.91(0.62-1.37)
≥ 29	37(4.0)	767(5.8)	1	1
Missing	43(4.6)	834(6.3)		
**Parity**				
0	15(1.6)	600(4.6)	1	1
1-2	224(24.0)	3754(28.5)	**2.38(1.41-4.05)**	**2.38 (1.40-4.08)**
3-4	248(26.6)	4086(31.0)	**2.41(1.43-4.32)**	**2.43(1.43-4.17)**
≥ 5	431(46.2)	4237(32.1)	**4.02(2.42-6.85)**	**3.19(1.84-5.48)**
Missing	14(1.5)	498(3.8)		

Table 3 summarizes the association between screening attendance and socioeconomic characteristics, sexual behavior, HIV status and HR-HPV infection. In the multivariate analysis no significant association was found between parity, age at first sexual intercourse, number of sexual partners and screening attendance. In contrast, a significant association was found between age, education and HIV status and screening attendances. More specifically, women aged 35–44 and women aged 45 and above had increased ORs of 1.53 (95% CI: 1.19-2.01) and 2.14 (95% CI: 2.90-1.56) respectively, for participating in the screening program in comparison with women who were aged 25–34. In addition, women with no formal education had increased OR of 1.96 (95% CI:1.23-3.17) for participating in the screening program in comparison with women who had attended secondary school. Finally, HIV positive women had an increased OR of 1.59 (95% CI: 1.14-2.25) for participating in the screening program in comparison with HIV negative women. No significant association was observed between HR-HPV infection and screening attendances.

**Table 3:** Socioeconomic characteristics, sexual behavior, HIV status and HR-HPV infection among screened and unscreened women

**Variable**	**Participated in the screening program N=890**	**Did not participate in the screening program N=845**	**OR (95% CI)**	Adjusted* OR (95% CI)
**Age**				
25-34	259(29.1)	332(39.3)	1	1
35-44	340(38.2)	304(36.0)	**1.43(1.14-1.18)**	**1.53(1.19-2.01)**
45-59	291(32.7)	209(24.7)	**1.78(1.39-2.29)**	**2.14(1.56-2.90)**
**Parity**				
0	79(8.9)	113(13.4)	1	1
1-2	307(34.5)	319(37.8)	1.38(0.99-1.91)	1.34(0.94-1.90)
3-4	293(33.0)	265(31.4)	**1.58(1.43-4.32)**	1.19(0.82-1.74)
≥ 5	210(23.6)	148(17.5)	**2.02(1.42-2.89)**	1.28(0.84-1.96)
**Educational Level**				
No formal education	74(8.3)	49(5.8)	1.50(0.97-2.23)	**1.96(1.23-3.17)**
Primary school	529(59.4)	516(61.1)	1.01(0.81-1.23)	1.06(0.50-1.51)
Sec school and above	287(32.2)	280(33.1)	1	1
**Age at 1**^**st**^**sexual intercourse**				
≤15	87(9.8)	89(10.5)	0.81(0.56-1.61)	0.75(0.49-1.15)
16-20	614(68.9)	600(71.0)	0.96(0.69-1.31)	0.83(0.63-1.09)
≥21	189(21.2)	156(18.5)	1	1
**No. of Sexual partners**				
≤1	334(37.5)	313(37.0)	1	1
2-3	376(42.2)	341(40.4)	1.03(0.83-1.29)	1.07(0.85-1.36)
≥4	180(20.2)	191(22.6)	0.94(0.68-1.15)	0.88(0.66-1.17)
**HIV status**				
Positive	107(12.0)	74(8.8)	**1.51(1.08-2.06)**	**1.59(1.14-2.25)**
Negative	694(78.0)	715(84.6)	**1**	
Missing	89(10.0)	56(6.6)		
**HR-HPV**				
Positive	128(17.8)	169(18.0)	1.06(0.84-1.36)	0.95(0.72-1.36)
Negative	634(75.8)	688(81.4.)	1	
Missing	57(6.4)	5(0.6)		

*We adjusted for age, parity, education, age of 1^st^ sexual intercourse, no of sexual partners, HIV status and HR-HPV.

Stratified analysis of HR-HPV and HIV infection among screened and unscreened women is summarized in Table 4. Women who attended screening were more likely to have HR-HPV infection as well as HIV infection in comparison with unscreened women.

**Table 4:** Stratified analysis of HR-HPV and HIV infection among screened and unscreened women

	**Participated in the screening program N (%)**	**Did not Participate in the screening program N (%)**	**OR (95% CI)**	aOR (95% CI)*
HR-HPV pos				
HIV pos	43(30.9)	34(24.3)	1.43(0.82-2.40)	1.90(1.06-5.74)
HIV neg	96(69.1)	106(75.7)	1	1
HR-HPV neg				
HIV pos	56(9.1)	40(6.2)	1.51(0.99-2.31)	1.42(0.81 - 3.76)
HIV neg	561(90.9)	606(93.8)	1	1

* adjusted for age.

## Discussion

In this study high age, low education, being widowed/separated, being married at a young age and being of high parity were associated with VIA positivity. Screening attendance was associated with being aged above 35, having no formal education and being HIV positive.

To our knowledge this is the first epidemiological study which has attempted to elicit risk factors for VIA positivity and screening attendance in Tanzania. Several factors may have affected the validity of the study findings and whether the sample of unscreened women can be considered a representative sub sample of the target group of women, who had never attended screening, may be questioned. However, to avoid selection bias and to assure as high representativity as possible, cluster randomisation was used to recruit women who had never participated in the screening program. When focusing on the screened women, the subsample appeared to be older than the full screened population. This may have biased the findings and resulted in an overestimation of the association between high age and screening attendance.

The VIA positivity rate among the 14,107 women screened at ORCI during the period 2002–2008 was 7%. In other cross-sectional studies, the proportion of VIA positive women varies from 2% to 16% [7,14,15]. It has been suggested that this observed heterogeneity in VIA positivity rates may be due to factors other than disease prevalence [16]. Hence, when discussing the validity of VIA as a screening method for cervical cancer there is concern about a lack of standardized test definition, differences in test providers skills, underlying prevalence of other sexually transmitted infections and lack of uniformity in the application of gold standard for disease definition [17]. These considerations should be taken into account in the interpretation of the VIA positivity rate reported in the present study.

VIA positivity was significantly correlated with being widowed/separated, being of high parity, being married at a young age and being of low education. Women who are separated or widowed may have higher number of lifetime sexual partners in comparison with married women and as number of lifetime sexual partners increases the risk of HPV infection they are more susceptible for developing precancerous lesions [18]. High parity is another well-known risk factor for precancerous lesions [19], this association is most likely due to repeated cervical trauma during consecutive births and hormonal adjustment during and after pregnancies which may create an entry point for the HPV virus. The found association between marriage at a young age and VIA positivity is consistent with a Nigerian study where marriage at early age was reported to be a risk factor for being VIA positive [20]. In line with this, a pooled analysis of case–control studies from eight developing countries have documented that early age at first sexual intercourse and early pregnancy are risk factors for cervical cancer [21]. The study further documented that the reported age at first sexual intercourse and age at first marriage correlated for 92% of the women [21]. These findings support a hypothesis that early age sexual intercourse is a risk factor for invasive cervical carcinoma. This hypothesis is further supported by a study conducted among married adolescent girls in urban centers in Kenya and Zambia which found that early marriage increases coital frequency, decreases condom use and virtually eliminates girls' ability to abstain from sex [22]. Furthermore, for women in some parts of the world, the behavior that puts them at risk of HIV infection and most likely HR-HPV infection is unprotected sex within marriage [23]. Although married girls are less likely than single girls to have multiple partners, this protective behavior may be outweighed by their greater exposure via unprotected sex with partners who have higher rates of infection, or if their husband has unprotected sex with other partners. Finally, the found association between VIA positivity and poor education may be explained by the fact that women who have not attended school are less informed about safe sex and condom use and are thereby at increased risk of acquiring a sexual transmitted infection, including HR-HPV infection.

In a low income setting, it may be argued that women who are widowed/separated, women who are having many children and women who are poorly educated share in common that they are at greater risk of being in a financial constraint situation and this may influence their health seeking behavior. A situation which together with the fact that they are at increased risk of HR-HPV infection place them at double jeopardy for developing cervical cancer. The association between low socioeconomic position and risk of developing cervical cancer has also been documented in a meta-analysis based on 57 studies [24]. The pooled analysis of the included studies found an increased risk of approximately 100% between high and low social classes for the development of invasive cervical cancer and an increased risk of approximately 60% for cervical dysplasia and carcinoma in situ. The increased risk was apparent in all regions, although it was stronger in America, Asia and Africa than in Europe.

HIV status was found to be significantly associated with screening attendance. Hence women who were HIV positive were more likely to participate in the screening program than HIV negative women were. This finding reflects the existence of a referral linkage between the HIV care and treatment program and the cervical cancer screening program in Dar es Salaam, where women who are tested HIV positive are encouraged to be screened for cervical cancer. The fact that HIV-infected women are more often infected with HR-HPV compared to HIV-uninfected women documents the need of such linkage. Based on our findings it may be argued that in countries with high HIV prevalence, the integration of cervical cancer into HIV care and treatment provides an ideal platform to reach HIV positive women who are at increased risk of developing cervical cancer. The cost effectiveness of this approach has also been documented in a Zambian study which showed that such integration allowed early detection of cervical cancer in HIV-infected women [25].

An association between HR-HPV infection and HIV infection was found. As the study design was cross sectional, it is not possible to determine whether the women acquired the HPV or the HIV infection first. One explanation behind the found association may be that HIV positive women are less likely to clear an HPV infection [9,26,27] and are more susceptibility to infection with multiple types of HPV [28-30]*.* In addition, it has also been suggested that HIV may be activating a dormant HPV infection and thus increase the risk of HPV-related disease [31].

## Conclusion

The findings from the present study indicate that women who are widowed/separated, of high parity, of low education and married at a young age are more likely to be VIA positive and thus at risk of developing cervical cancer. The study further documents that a referral linkage between the HIV care and treatment program and the cervical cancer screening program is in place in the setting studied where HIV positive women were more likely to participate in the screening program than HIV negative women were. To increasingly reach socially deprived women, cervical cancer screening awareness campaigns should be performed and integrating cervical cancer screening in existing reproductive health care programs should be considered.

## Competing interests

KKS received lecture fees, advisory board fees, and research grants through her institution from Merck and Sanofi Pasteur MSD. The author(s) declare that they have no competing interests.

## Authors’ contributions

KC participated in the conception, design, and implementation of the study, statistical analysis, interpretation and drafting of manuscript. KKS and RV participated in developing the study design, the implementation and the interpretation of the findings. DM, MJ, NT participated in developing the study design and assisted with implementation of the study and IT performed the HPV analyses. All authors read and approved the final manuscript.

## Pre-publication history

The pre-publication history for this paper can be accessed here:

http://www.biomedcentral.com/1471-2458/12/1055/prepub

## References

[B1] Arbyn M Castellsague X de Sanjose S Bruni L Saraiya M Bray F Ferlay J Worldwide burden of cervical cancer in 2008 Ann Oncol 2011 22 12 2675 2686 10.1093/annonc/mdr015 21471563

[B2] Sankaranarayanan R Budukh AM Rajkumar R Effective screening programmes for cervical cancer in low- and middle-income developing countries Bull World Health Organ 2001 79 10 954 962 11693978 PMC2566667

[B3] CCA Progress in Cervical Cancer Prevention: The CCA Report Card 2012 Cervical Cancer Action Available from: http://www.cervicalcanceraction.org/pubs/CCA_reportcard_low-res.pdf

[B4] CDC United States Cancer Statistics: 1999–2007 Incidence and Mortality Web-based Report 2010 Atlanta: Department of Health and Human Services; Centers for Disease Control and Prevention; National Cancer Institute

[B5] Denny L Kuhn L Pollack A Wright TC Jr Direct visual inspection for cervical cancer screening: an analysis of factors influencing test performance Cancer 2002 94 6 1699 1707 10.1002/cncr.10381 11920531

[B6] Sankaranarayanan R Wesley RS A practical manual on visual screening for cervical neoplasia 2003 Lyon, France: International Agency for Research on Cancer Technical publication

[B7] Ngoma T Muwonge R Mwaiselage J Kawegere J Bukori P Sankaranarayanan R Evaluation of cervical visual inspection screening in Dar es Salaam, Tanzania Int J Gynaecol Obstet 2010 109 2 100 104 10.1016/j.ijgo.2009.11.025 20152973

[B8] Sankaranarayanan R Rajkumar R Esmy PO Fayette JM Shanthakumary S Frappart L Thara S Cherian J Effectiveness, safety and acceptability of 'see and treat' with cryotherapy by nurses in a cervical screening study in India Br J Cancer 2007 96 5 738 743 10.1038/sj.bjc.6603633 17311015 PMC2360066

[B9] Kahesa C Mwaiselage J Wabinga HR Ngoma T Kalyango JN Karamagi CA Association between invasive cancer of the cervix and HIV-1 infection in Tanzania: the need for dual screening BMC Publ Health 2008 8 262 10.1186/1471-2458-8-262 PMC2527006 18664298

[B10] UNAIDS Report on Global Aids Epidermic 2008 Available from: http://www.unaids.org/en/dataanalysis/knowyourepidemic/epidemiologypublications/2008reportontheglobalaidsepidemic/

[B11] National Bureau of Statistics Tanzania National House Hold Survey 2007 Dar es Salaam

[B12] WHOomprehesive cervical cancer control: A guide to essential practice 2006 Geneva: World Health Organization 25642554

[B13] Lyamuya EF Aboud S Urassa WK Sufi J Mbwana J Ndugulile F Massambu C Evaluation of simple rapid HIV assays and development of national rapid HIV test algorithms in Dar es Salaam, Tanzania BMC Infect Dis 2009 9 19 10.1186/1471-2334-9-19 19226452 PMC2650699

[B14] Mayaud P Gill DK Weiss HA Uledi E Kopwe L Todd J Ka-Gina G Grosskurth H Hayes RJ Mabey DC The interrelation of HIV, cervical human papillomavirus, and neoplasia among antenatal clinic attenders in Tanzania Sex Transm Infect 2001 77 4 248 254 10.1136/sti.77.4.248 11463923 PMC1744347

[B15] Muwonge R Walter SD Wesley RS Basu P Shastri SS Thara S Mbalawa CG Sankaranarayanan R Assessing the gain in diagnostic performance when two visual inspection methods are combined for cervical cancer prevention J Med Screen 2007 14 3 144 150 10.1258/096914107782066158 17925087

[B16] Vedantham H Silver MI Kalpana B Rekha C Karuna BP Vidyadhari K Mrudula S Ronnett BM Vijayaraghavan K Ramakrishna G Determinants of VIA (Visual Inspection of the Cervix After Acetic Acid Application) positivity in cervical cancer screening of women in a peri-urban area in Andhra Pradesh, India Cancer Epidemiol Biomarkers Prev 2010 19 5 1373 1380 10.1158/1055-9965.EPI-09-1282 20447927 PMC2913449

[B17] Mahe C Gaffikin L Screening test accuracy studies: how valid are our conclusions? Application to visual inspection methods for cervical screening Cancer Causes Control 2005 16 6 657 666 10.1007/s10552-005-0296-4 16049804

[B18] Ho GY Studentsov YY Bierman R Burk RD Natural history of human papillomavirus type 16 virus-like particle antibodies in young women Cancer Epidemiol Biomarkers Prev 2004 13 1 110 116 10.1158/1055-9965.EPI-03-0191 14744741

[B19] Almonte M Ferreccio C Gonzales M Delgado JM Buckley CH Luciani S Robles SC Winkler JL Tsu VD Jeronimo J Risk factors for high-risk human papillomavirus infection and cofactors for high-grade cervical disease in Peru Int J Gynecol Cancer 2011 21 9 1654 1663 10.1097/IGC.0b013e3182288104 21892094

[B20] Ogunbowale T Lawoyin TO Cervical cancer risk factors and predictors of cervical dysplasia among women in south-west Nigeria Aust J Rural Health 2008 16 6 338 342 10.1111/j.1440-1584.2008.01013.x 19032205

[B21] Louie KS de Sanjose S Diaz M Castellsague X Herrero R Meijer CJ Shah K Franceschi S Munoz N Bosch FX Early age at first sexual intercourse and early pregnancy are risk factors for cervical cancer in developing countries Br J Cancer 2009 100 7 1191 1197 10.1038/sj.bjc.6604974 19277042 PMC2670004

[B22] Clark S Early marriage and HIV risks in sub-Saharan Africa Stud Fam Plann 2004 35 3 149 160 10.1111/j.1728-4465.2004.00019.x 15511059

[B23] Hirsch JS Higgins J Bentley ME Nathanson CA The social constructions of sexuality: marital infidelity and sexually transmitted disease-HIV risk in a Mexican migrant community Am J Public Health 2002 92 8 1227 1237 10.2105/AJPH.92.8.1227 12144974 PMC1447220

[B24] Parikh S Brennan P Boffetta P Meta-analysis of social inequality and the risk of cervical cancer Int J Cancer 2003 105 5 687 691 10.1002/ijc.11141 12740919

[B25] Mwanahamuntu MH Sahasrabuddhe VV Kapambwe S Pfaendler KS Chibwesha C Mkumba G Mudenda V Hicks ML Vermund SH Stringer JS Advancing cervical cancer prevention initiatives in resource-constrained settings: insights from the Cervical Cancer Prevention Program in Zambia PLoS Med 2011 8 5 e1001032 10.1371/journal.pmed.1001032 21610859 PMC3096609

[B26] Chirenje ZM Loeb L Mwale M Nyamapfeni P Kamba M Padian N Association of cervical SIL and HIV-1 infection among Zimbabwean women in an HIV/STI prevention study Int J STD AIDS 2002 13 11 765 768 10.1258/095646202320753727 12437897

[B27] Parham GP Sahasrabuddhe VV Mwanahamuntu MH Shepherd BE Hicks ML Stringer EM Vermund SH Prevalence and predictors of squamous intraepithelial lesions of the cervix in HIV-infected women in Lusaka, Zambia Gynecol Oncol 2006 103 3 1017 1022 10.1016/j.ygyno.2006.06.015 16875716 PMC2748907

[B28] Hawes SE Critchlow CW Faye Niang MA Diouf MB Diop A Toure P Aziz Kasse A Dembele B Salif Sow P Coll-Seck AM Increased risk of high-grade cervical squamous intraepithelial lesions and invasive cervical cancer among African women with human immunodeficiency virus type 1 and 2 infections J Infect Dis 2003 188 4 555 563 10.1086/376996 12898443

[B29] Sun XW Kuhn L Ellerbrock TV Chiasson MA Bush TJ Wright TC Jr Human papillomavirus infection in women infected with the human immunodeficiency virus N Engl J Med 1997 337 19 1343 1349 10.1056/NEJM199711063371903 9358128

[B30] Vuyst HD Ndirangu G Moodley M Tenet V Estambale B Meijer CJ Snijders PJ Clifford G Franceschi S Prevalence of human papillomavirus in women with invasive cervical carcinoma by HIV status in Kenya and South Africa Int J Cancer 2011 131 4 949 955 21960453 10.1002/ijc.26470

[B31] Theiler RN Farr SL Karon JM Paramsothy P Viscidi R Duerr A Cu-Uvin S Sobel J Shah K Klein RS High-risk human papillomavirus reactivation in human immunodeficiency virus-infected women: risk factors for cervical viral shedding Obstet Gynecol 2010 115 6 1150 1158 10.1097/AOG.0b013e3181e00927 20502284

